# Mother’s warmth from maternal genes: genomic imprinting of brown adipose tissue

**DOI:** 10.1093/emph/eoad031

**Published:** 2023-09-29

**Authors:** Lynn Ayache, Aiden Bushell, Jessica Lee, Iiro Salminen, Bernard Crespi

**Affiliations:** Department of Biological Sciences, Simon Fraser University, 8888 University Drive, Burnaby, British Columbia V5A 1S6, Canada; Department of Biological Sciences, Simon Fraser University, 8888 University Drive, Burnaby, British Columbia V5A 1S6, Canada; Department of Biological Sciences, Simon Fraser University, 8888 University Drive, Burnaby, British Columbia V5A 1S6, Canada; Department of Biological Sciences, Simon Fraser University, 8888 University Drive, Burnaby, British Columbia V5A 1S6, Canada; Department of Biological Sciences, Simon Fraser University, 8888 University Drive, Burnaby, British Columbia V5A 1S6, Canada

**Keywords:** genomic imprinting, brown adipose tissue, genomic conflict, obesity, thermoregulation

## Abstract

**Background and objectives:**

Brown adipose tissue (BAT) plays key roles in mammalian physiology, most notably with regard to thermoregulation in infants and juveniles. Previous studies have suggested that intragenomic conflict, in the form of genomic imprinting, mediates BAT thermogenesis, because it represents a public good for groups of siblings, or a mother with her offspring, who huddle together to conserve warmth. By this hypothesis, maternally expressed imprinted genes should promote BAT, while paternally expressed genes should repress it.

**Methodology:**

We systematically searched the literature using two curated lists of genes imprinted in humans and/or mice, in association with evidence regarding effects of perturbation to imprinted gene expression on BAT development or activity.

**Results:**

Overall, enhanced BAT was associated with relatively higher expression of maternally expressed imprinted genes, and relatively lower expression of paternally expressed imprinted genes; this pattern was found for 16 of the 19 genes with sufficient information for robust ascertainment (Binomial test, *P* < 0.005, 2-tailed).

**Conclusions and implications:**

These results support the kinship theory of imprinting and indicate that future studies of BAT, and its roles in human health and disease, may usefully focus on effects of imprinted genes and associated genomic conflicts.

‘How we need another soul to cling to, another body to keep us warm’.

Sylvia Plath, Unabridged Journals of Sylvia Plath, 1982.

## 1. Introduction

Brown adipose tissue (BAT), which generates body heat from uncoupled mitochondrial respiration, plays central roles in mammalian physiology with regard to thermoregulation [1], release of adipokines that act on other target tissues [2], and regulation of body weight and adiposity [3]. Discerning the mechanisms whereby BAT mediates these activities requires an understanding of the genetic bases of BAT development and function, as well as the adaptive significance of BAT activity in different physiological and evolutionary contexts. Over the past decade, the study of BAT genetics and physiology in humans has taken on increasing importance, given the demonstrated effects of this tissue in risks of obesity [4] and reproductive dysfunction [5].

Recent studies of BAT have elucidated its adaptive significance with regard to its anatomical positioning [6], life-stage trajectories [7], gender differences [8] and ancestral climatic variation influencing current human diversity in its prevalence and function (e.g. [9]). These studies have demonstrated that BAT exhibits a set of age-specific, sex-specific and climate-specific adaptive functions related to tradeoffs involving body-heat production, protection from hypothermia, growth, and deposition of white fat, such that survival and reproduction are conditionally optimized under particular patterns of BAT tissue levels and activation [1, 3, 7–9]. Evolutionary considerations have also provided notable insights into adaptive genetic and geographic variation in BAT activity among human groups, as well as helping to show how recent environmental changes, including widespread use of efficient heating systems, increased food intake, reduced physical activity and migration to regions with different thermal regimes, have apparently generated BAT-related evolutionary mismatches with ancestral environments that contribute to the rising prevalence of obesity and associated diseases [1].

Research on the genetic bases and adaptive significance of BAT has focused on elucidating the sets of genes and pathways that regulate its development and metabolic activity [10]. This work has proceeded predominantly from genetic, molecular and physiological perspectives, and has yet to substantially incorporate insights from evolutionary biology into the hypotheses addressed and genes and mechanisms chosen for analysis. A primary consideration from evolutionary theory in this context, is that BAT, and its genetic control, originated with the evolution of early mammals, especially among the first eutherians [11, 12]. Such ancestral mammals were characterized by small size, endothermy, production of offspring in broods, extensive maternal care, and the presence of genomic imprinting, an epigenetic mechanism whereby the maternal or paternal allele at a locus is silenced (‘imprinted’) [13].

Evolutionary theory, supported by extensive empirical evidence, indicates that paternal allele expression from imprinted genes is associated with relatively ‘selfish’ effects on relatives (typically, siblings or the mother), whereas maternal allele expression is linked with cooperation [14]. As described by Haig [15, 16], the BAT-derived heat produced by newborn and juvenile individuals in broods of mammals that huddle together (such as small rodents) represents cooperative use of a shared resource. In such situations, the production of metabolic heat, as by BAT, is expected from evolutionary considerations to come partially under the control of imprinted genes, with maternally expressed genes promoting greater heat production (which benefits the brood and the mother in terms of energy efficiency and group-member survival), and paternally expressed imprinted genes exerting effects that selfishly reduce BAT-based heat-producing mechanisms (which benefits a brood member in terms of higher energy availability for growth and survival). The predicted outcomes are thus that maternally expressed imprinted genes should have effects that favor BAT thermogenesis, and that paternally expressed imprinted genes should mediate effects that reduce it.

Haig [15] described how these predictions, that maternally expressed imprinted genes favor BAT, while paternally expressed ones disfavor it, were met, for the genes *DLK1* and *NDN*, and two genes at the complex *GNAS* locus , from several experimental studies of mice. He also suggested that ‘future studies will test whether this pattern is maintained at other imprinted loci’ [15]. The purpose of this article is to evaluate Haig’s prediction, using data from the documented imprinted genes in humans and mice. These data permit tests of a prediction of the theory of genomic imprinting in this context, provide opportunities for new insights into the genetic control of BAT and, given such findings, indicate new possibilities for pharmacological manipulation of BAT development and function. The specific prediction under test is that, for imprinted genes that are associated with BAT levels or activity, increased expression of maternally expressed imprinted genes, and reduced expression of paternally expressed imprinted genes, will enhance BAT; by contrast, decreased expression of maternally expressed imprinted genes, and increased expression of paternally expressed imprinted genes, will reduce it.

## 2. METHODS

Lists of imprinted protein-coding or long non-coding RNA genes were derived from the Geneimprint Imprinted Gene Database (https://www.geneimprint.com/site/genes-by-species) and the Imprinted Gene and Parent of Origin Effect Database (https://corpapp.otago.ac.nz/gene-catalogue), both updated as of 2022. Only genes validated as imprinted were included, and ‘predicted’ imprinted genes were excluded. Any discrepancies between the two databases were resolved using literature searches on the putatively imprinted gene in question, and all genes listed as imprinted, and associated with brown fat in the literature searches described below, were also verified as imprinted, in all or specific tissues examined, using the relevant studies from the primary literature.

All genes thereby listed as imprinted in humans, mice, or both in either or both databases were subject to searches in PubMed and Web of Science using the search terms: ‘gene name’ (and relevant synonyms and protein product names) AND ‘brown adipose’, and ‘gene name’ AND ‘brown fat’, to ascertain the presence of links of specific imprinted genes with BAT levels and activity. The direction of imprinted gene expression effects on BAT was determined via the results of gene knockout, knockin, and gene or protein expression level studies, where such data was available; for example, knockout in mice of a BAT-associated paternally expressed gene, that makes gene expression maternally biased, would be predicted to increase BAT.

Supplementary Tables 1 and 2 present the detailed results as regards genes searched and results returned.

BAT levels and activity were ascertained in the relevant studies by several means including measurement of tissue mass, measurement of temperature in BAT-associated anatomical regions, quantification of expression levels from key BAT-associated genes (e.g. Uncoupling Protein 1, *UCP-1*) or other approaches as specified.

For genes that show differences in imprinting patterns between humans and mice (e.g. imprinted in one species but not in the other), the link between expression pattern and BAT effects needed to be shown for the gene within the species subject to imprinting. When imprinting was not ubiquitous across the body, the specific tissues where imprinting was reported needed to, at least in principle, be relevant to BAT development or function; for example, studies of a gene that is imprinted only in extraembryonic tissues (the placenta), such as *XIST* [17], would not be relevant to the hypothesis addressed.

The null expectation was that perturbations to imprinted gene expression had effects on brown fat that went in directions that were independent of paternal versus maternal gene expression. By contrast, the hypothesis under test predicted that increases in maternal gene expression, and decreases in paternal gene expression, lead to increases in brown fat extent or activity, and that decreases in maternal gene expression, and increases in paternal gene expression, lead to decreases in brown fat extent or activity.

## 3. RESULTS

Overall, 19 imprinted genes fit the criteria for inclusion in the analysis, in showing clear evidence of directional effects on BAT activity or development, from experimental alterations to expression (Table 1 and Supplementary Tables 1 and 2). Of the 19 genes, 16 fit the predictions, and 3 did not (Binomial test, *P* = 0.0044, 2-tailed). The 16 genes were about equally distributed between those expressed paternally (seven genes) and maternally (nine genes), and the three latter genes were all maternally expressed.

**Table 1.** Evidence salient to the hypothesis that maternally expressed imprinted genes favor the development or activity of brown adipose tissue, while paternally expressed imprinted genes reduce it

Gene	Expressed allele	Phenotypes	Notes	References
+ *CD81*	Maternal	Expression required for beige fat production after exposure to cold in mice	Loss of expression causes impaired beiging and obesity	[18]
+ *CDKN1C*	Maternal	Doubled expression leads to extremely thin phenotype due to increased energy consumption and increased browning of white adipose tissue	*CDKN1C* deletion also associated with increased expression for genes involved in muscle differentiation	[19]
+ *DCN*	Maternal (mice)	*UCP1* mRNA expression reduced in BAT of knockout mice on high fat diet, and glucose tolerance reduced	Altered fat metabolism in knockouts	[20]
+ *DIO3*	Paternal	Expression inhibits brown adipocyte differentiation; increased expression leads to reduced thermogenesis in offspring; inactivation of *DIO3* leads to early expression of BAT genes	Gene product breaks down thyroid hormones, regulating fetal BAT development	[21–23]
+ *DIO3OS*	Maternal	Knockdown of *DIO3OS* leads to reduced adipogenic differentiation	Antisense RNA to *DIO3*, typical function is dependant on the reduced amount of *DIO3* expression, due to RNA interference between *DIO3* and *DIO3OS*	[21]
+ *DLK1*	Paternal	*DLK1* inhibits brown adipocyte differentiation; overexpression leads to increased lethality via hypothermia, and reduced thermogenesis in juveniles	*DLK1* may act upstream of *PRDM16* and *PPARγ* in adipogenesis	[24]
+ *GATM*	Maternal (mice, not humans)	Mouse knockouts show reduced β3-adrenergic activity and reduced energy expenditure, with impaired BAT activity and lower body temperature	Depletion of creatine in adipocytes inhibits thermogenesis in knockouts	[25, 26]
+ *GNAS*	Maternal	Reduced thermogenesis with deletion, with brown adipocytes resembling white adipocytes	*GNAS* takes part in a signal cascade regulating non-shivering thermogenesis upon exposure to cold	[27, 28]
+ *GNAS*-*AS/NESPAS*	Paternal	Deletion leads to a thin phenotype	Paternally expressed *NESPAS* inhibits *GNAS* expression from paternal allele in brown fat	[29]
+ *GNASXL*	Paternal	Mice with paternal deletion are lean and hypermetabolic, with increases SNS stimultion of BAT tissue and higher conversion of white to beige adipose tissue and higher body temperature	Both *GNAS* and *GNASXL* are expressed in adipose tissues, *GNASXL* inhibits cAMP signaling in brown adipocytes	[30–33]
+ *GPR1*	Paternal	*GPR1* knockout in mice leads to increased *UCP1* expression and increased BAT weight under high fat diet	Gene product chemerin is adipokine with metabolic effects	[34, 35]
+ *GRB10*	Maternal (in body, not brain)	Highly expressed in BAT, induced by cold in WAT; knockouts in fat, in mice, lead to impaired thermogenesis and obesity	*G* *RB* *10* downregulates mTOR1 signaling cascade (involved in activation of lipolysis and non-shivering thermogenesis)	[36]
+ *H19*	Maternal	*H19* levels increase in BAT under cold activation and decrease in obese individuals; overexpression increases BAT activity and insulin sensitivity	*H19* recruits *MBD1* complex that inactivates paternally expressed imprinted genes in BAT	[37, 38]
+ *Mir-337*	Maternal	Over-expression in brown pre-adipocytes increases brown fat and mitochondrial markers in mice	*Mir-337* enhances production of brown adipocytes by inhibiting *TWIST1*. Lower *Mir-337* is associated with higher obesity in humans	[39]
+ *NDN*	Paternal	Suppression of necdin expression increases BAT differentiation and adipogenesis; *NDN* also impairs BAT differentiation in vitro	*N* *DN* inhibits *PPARγ1* expression in adipogenesis via interaction with E2F4/DP-1 protein complex	[40–42]
+ *NNAT*	Paternal	Silencing of *NNAT* in murine primary adipocyte cultures is associated with higher UCP1 expression and other indicators of browning	*NNAT* acts as negative regulator of browning gene expression program	[43]
– *SLC22A3*	Maternal (mice and humans)	*SLC22A3 (OCT3)* knockout mice show increased beiging of white fat tissue		[44]
– *PDE10A*	Maternal (mice)	Pharmacological inactivation of *PDE10* increases BAT activity and energy expenditure, and enhances insulin sensitivity		[45]
− *RB1*	Maternal in humans, not imprinted in mice	*RB1* knockdown in preadipocytes leads to increased *UCP1* and *PRDM16* mRNA; transient knockdown in mature adipocytes has no effect	In visceral and subcutaneous fat, higher *RB1* levels are associated with lower body mass index and lower insulin resistance	[46, 47]

Genes supporting the hypothesis (with a ‘+’) are listed first, alphabetically, and genes that do not support it (with a ‘−’) follow.

## 4. CONCLUSIONS

The hypothesis that maternally expressed imprinted genes tend to favor the development and activity of BAT, whereas paternally expressed imprinted genes disfavor it, was statistically supported by the available data. The main implications of this findings are 2-fold.

First, these results support the kinship theory of genomic imprinting, in that BAT activity represents a common good, shared among siblings in a mother’s brood, that differentially benefits the mother and maternal genes in her offspring [14–16]. The benefits of this shared good have been demonstrated to be substantial: for example, by huddling, rodent pups can increase their metabolic efficiency by 40–65%, thereby reducing nutritive maintenance requirements and substantially increasing rates of growth [48–52].

Small, brood-producing mammals share warmth mainly or exclusively in a huddle of infants or juveniles. This selective situation differs considerably from that in humans, where mother-offspring dyads are the usual situation. Heat production in human infants is limited to non-shivering thermogenesis, with BAT representing about 10% of body weight at birth [53]. The primary benefit to mothers, and maternal genes (in the brood and in other maternal relatives, such as maternal-lineage allomothers), of increased heat production by infants would be reduced energetic demands imposed on the mother, at a time when such demands are especially elevated due to the high energetic costs of lactation [54]. In mice, mothers indeed reduce their own BAT activity by around 50% during lactation (compared to non-reproducing females), in part to save energy for the production of milk [55].

The primary benefits to paternal genes of reduced BAT levels and activity in an individual are expected to be enhanced growth and increased deposition of white fat. For example, higher expression of the paternally expressed imprinted gene *DIO3* is associated with diet-induced obesity in mice [21], and higher expression of the paternally expressed imprinted gene *DLK1* is associated with higher levels of white fat in humans [56].

The second primary implication of this article is that it helps to consolidate a new conceptual and molecular-genetic framework for understanding BAT thermogenesis, centered on important roles for imprinted genes. Genes subject to imprinting are dosage-sensitive, which makes them relatively amenable to experimental and therapeutic manipulations, as well as exhibiting forms of intragenomic conflict, whereby loci or transcripts (e.g. at the *DIO3* and *GNAS* loci) exhibit opposing effects on BAT development or function. These properties of imprinted genes, and their increased vulnerability to dysregulation by genetic and epigenetic alterations, make them especially important systems for understanding the impacts of BAT on human health and disease. Such impacts are considerable: BAT activity substantially enhances cardiovascular and metabolic health, including effects on risks of type 2 diabetes, dyslipidemia, coronary artery disease, and hypertension, with differentially high benefits to individuals who are overweight [57, 58]. As such, activation of BAT may mitigate many of the deleterious health effects of obesity, even if weight levels are not reduced. Altered expression of specific imprinted genes is also known to exert notable effects on metabolic health: thus, higher expression of the maternally expressed imprinted genes *CDKN1C* and *H19*, and lower expression of the paternally expressed imprinted gene *DLK1*, each protects against diet-induced obesity in mice [19, 37, 56].

This study is subject to several limitations that constrain its generality and methodological robustness. For example, tissue-specific imprinting has yet to be studied using BAT-expressed transcripts, such that the results presented here come predominantly from genes that are imprinted across many or all tissues studied. Some imprinted genes have also been subject to much more study than others, within and across taxa; for example, 6 of the 19 genes in Table 1 have been analyzed for BAT-related effects in only a single publication. The distribution of taxa analyzed for genomic imprinting effects is also limited and uneven, with most research effort devoted to mice and humans, less work on rats, sheep, pigs, and cattle and few additional eutherian taxa analyzed in many studies or much detail (Geneimprint database, https://www.geneimprint.com/site/genes-by-species). Between humans and mice, conservation of imprinting status is low to moderate overall (e.g. 16% across all imprinted genes in [Fig. 2 in [59]]. Such limitations should compel further studies on the roles of imprinted genes in BAT-related thermogenesis and energetics across different eutherian taxa, including species that do not exhibit huddling of juveniles or extended mother-infant contact, and thus might not be expected to show genomic imprinting effects on BAT levels and activity.

It also remains unclear why *SLC22A3*, *PDE10A* and *RB1* show patterns that are opposite to those predicted. Such findings may be due in part to pleiotropy, whereby evolved imprinting effects for these genes exhibit predominant functions in other contexts (e.g. for *RB1*, which regulates passage through the cell cycle across many tissues, and is imprinted in humans and related primates but, unusually, not in other mammals [60]). Studies of imprinted gene expression in BAT, and how imprinted genes mediate thermoregulation of infants and young juveniles, are most germane to further tests of the hypothesis that maternally expressed imprinted genes differentially drive the shared warmth, and common good, of huddling together.

## Supplementary Material

eoad031_suppl_Supplementary_Table_S1

eoad031_suppl_Supplementary_Table_S2
