# Supplementary material for: Mother’s warmth from maternal genes: genomic imprinting of brown adipose tissue
Source: Evol Med Public Health. 2023 Sep 29;11(1):379–85. doi: 10.1093/emph/eoad031 (PMC10621903; doi:10.1093/emph/eoad031)
Supplement: eoad031_suppl_Supplementary_Table_S1 [file eoad031_suppl_supplementary_table_s1.docx]

Supplementary Table 1. Results returned from searches using the Geneimprint Imprinted Gene Database

| **Human** | | | | |
| --- | --- | --- | --- | --- |
| **Gene name** | **Expressed allele** | **WOS returns** brown fat; brown adipose | **PM returns** brown fat; brown adipose | **Meeting criteria (see citations in Table 1)** |
| ADTRP | Maternal | 1; 1 | 0; 1 |  |
| AIM1 | Paternal | 0; 0 | 0; 0 |  |
| ANO1 | Maternal | 0; 0 | 0; 0 |  |
| ATP10A | Maternal | 0; 0 | 0; 0 |  |
| ATP5F1EP2 | Maternal | 0; 0 | 0; 0 |  |
| CALCR | Maternal | 0; 0 | 0; 0 |  |
| CCDC71L | Paternal | 0; 0 | 0; 0 |  |
| CDKN1C | Maternal | 2; 2 | 1; 2 | x |
| CMTM1 | Paternal | 0; 0 | 0; 0 |  |
| COPG2 | Paternal | 0; 0 | 0; 0 |  |
| COPG2IT1 | Paternal | 0; 0 | 0; 0 |  |
| CPA4 | Maternal | 0; 0 | 0; 0 |  |
| CTNNA3 | Maternal | 0; 0 | 0; 0 |  |
| DIO3 | Paternal | 7; 13 | 5; 9 | x |
| DIO3OS | Maternal | 1; 2 | 1; 2 | x |
| DIRAS3 | Paternal | 0; 0 | 0; 0 |  |
| DLGAP2 | Paternal | 0; 0 | 0; 0 |  |
| DLK1 | Paternal | 5; 8 | 4; 13 | x |
| DLX5 | Maternal | 0; 2 | 0; 1 |  |
| DNMT1 | Paternal | 6; 7 | 2; 4 |  |
| DSCAM | Paternal | 0; 0 | 0; 0 |  |
| ERAP2 | Paternal | 0; 1 | 0; 0 |  |
| FAM50B | Paternal | 0; 0 | 0; 0 |  |
| FOXF1 | Maternal | 1; 1 | 0; 0 |  |
| GABRA5 | Paternal | 0; 0 | 0; 0 |  |
| GABRB3 | Paternal | 0; 0 | 0; 0 |  |
| GABRG3 | Paternal | 0; 0 | 0; 0 |  |
| GDAP1L1 | Paternal | 0; 0 | 0; 0 |  |
| GLI3 | Paternal | 2; 2 | 1; 2 |  |
| GLIS3 | Paternal | 0; 0 | 0; 0 |  |
| GNASAS | Paternal | 0; 0 | 0; 0 |  |
| GPR1 | Paternal | 2; 2 | 0; 1 | x |
| H19 | Maternal | 4; 4 | 2; 1 | x |
| HECW1 | Paternal | 0; 0 | 0; 0 |  |
| HOXA4 | Maternal | 1; 1 | 2; 0 |  |
| HTR2A | Maternal | 1; 1 | 0; 1 |  |
| HYMAI | Paternal | 0; 0 | 0; 0 |  |
| IGF2 | Paternal | 4; 5 | 1; 4 |  |
| IGF2AS | Paternal | 0; 0 | 0; 0 |  |
| INPP5F V2 | Paternal | 0; 0 | 0; 0 |  |
| INS | Paternal | 16; 20 | 0; 4 |  |
| IRAIN | Paternal | 0; 0 | 0; 0 |  |
| KCNK9 | Maternal | 0; 0 | 0; 1 |  |
| KCNQ1 | Maternal | 0; 0 | 0; 0 |  |
| KCNQ1DN | Maternal | 0; 0 | 0; 0 |  |
| KCNQ1OT1 | Paternal | 1; 1 | 0; 0 |  |
| KLF14 | Maternal | 0; 1 | 0; 0 |  |
| L3MBTL1 | Paternal | 0; 0 | 0; 0 |  |
| LIN28B | Paternal | 0; 0 | 0; 0 |  |
| LRRTM1 | Paternal | 0; 0 | 0; 0 |  |
| MAGEL2 | Paternal | 1; 1 | 0; 0 |  |
| MAGI2 | Maternal | 0; 0 | 0; 0 |  |
| MCTS2 | Paternal | 0; 0 | 0; 0 |  |
| MEG3 | Maternal | 3; 2 | 0; 1 |  |
| MEG8 | Maternal | 0; 0 | 0; 0 |  |
| MEST | Paternal | 33; 39 | 0; 3 |  |
| MESTIT1 | Paternal | 0; 0 | 0; 0 |  |
| MIMT1 | Paternal | 0; 0 | 0; 0 |  |
| MIR296 | Paternal | 0; 0 | 0; 0 |  |
| MIR298 | Paternal | 0; 0 | 0; 0 |  |
| MIR371A | Paternal | 0; 0 | 0; 0 |  |
| MKRN3 | Paternal | 0; 1 | 0; 0 |  |
| NAA60 | Maternal | 0; 0 | 0; 0 |  |
| NAP1L5 | Paternal | 0; 0 | 0; 0 |  |
| NDN | Paternal | 1; 0 | 0; 0 | x |
| NLRP2 | Maternal | 0; 0 | 0; 1 |  |
| NNAT | Paternal | 1; 2 | 0; 3 | x |
| NPAP1 | Paternal | 0; 0 | 0; 0 |  |
| NTM | Maternal | 0; 1 | 0; 0 |  |
| OSBPL5 | Maternal | 0; 0 | 0; 0 |  |
| PARD6G | Maternal | 0; 0 | 0; 0 |  |
| PEG10 | Paternal | 1; 0 | 0; 0 |  |
| PEG13 | Paternal | 0; 0 | 0; 0 |  |
| PEG3 | Paternal | 0; 0 | 0; 0 |  |
| PEG3-AS1 | Paternal | 0; 0 | 0; 0 |  |
| PHLDA2 | Maternal | 0; 0 | 0; 0 |  |
| PLAGL1 | Paternal | 1; 1 | 0; 0 |  |
| PON1 | Maternal | 2; 2 | 0; 2 |  |
| PPP1R9A | Maternal | 0; 0 | 0; 0 |  |
| PRR25 | Paternal | 0; 0 | 0; 0 |  |
| PSIMCT-1 | Paternal | 0; 0 | 0; 0 |  |
| PWAR6 | Paternal | 0; 0 | 0; 0 |  |
| PWCR1 | Paternal | 0; 0 | 0; 0 |  |
| PXDC1 | Paternal | 0; 0 | 0; 0 |  |
| RASGRF1 | Paternal | 0; 0 | 0; 0 |  |
| RB1 | Maternal | 9; 14 | 1; 6 | x |
| RBP5 | Maternal | 1; 1 | 0; 1 |  |
| RHOBTB3 | Paternal | 0; 0 | 0; 0 |  |
| RNU5D-1 | Paternal | 0; 0 | 0; 0 |  |
| RTL1 | Paternal | 0; 0 | 0; 0 |  |
| SANG | Paternal | 98; 100 | 0; 5 |  |
| SDHD | Paternal | 0; 2 | 0; 4 |  |
| SGCE | Paternal | 1; 0 | 0; 0 |  |
| SGK2 | Paternal | 0; 2 | 0; 0 |  |
| SLC22A18 | Maternal | 0; 0 | 0; 0 |  |
| SLC22A18AS | Maternal | 0; 0 | 0; 0 |  |
| SLC22A2* | Maternal | 0; 0 | 0; 1 |  |
| SLC22A3* | Maternal | 2; 2 | 0; 0 | x |
| SMOC1 | Maternal | 0; 0 | 0; 0 |  |
| SNORD107 | Paternal | 0; 0 | 0; 0 |  |
| SNORD108 | Paternal | 0; 0 | 0; 0 |  |
| SNORD109A | Paternal | 0; 0 | 0; 0 |  |
| SNORD109B | Paternal | 0; 0 | 0; 0 |  |
| SNORD113-1 | Maternal | 0; 0 | 0; 0 |  |
| SNORD114-1 | Maternal | 0; 0 | 0; 0 |  |
| SNORD115 | Paternal | 0; 0 | 0; 0 |  |
| SNORD115-48 | Paternal | 0; 0 | 0; 0 |  |
| SNORD116 | Paternal | 0; 0 | 0; 0 |  |
| SNORD64 | Paternal | 0; 0 | 0; 0 |  |
| SNRPN | Paternal | 1; 0 | 0; 0 |  |
| SNURF | Paternal | 0; 0 | 0; 0 |  |
| ST8SIA1 | Paternal | 0; 0 | 0; 0 |  |
| SVOPL | Maternal | 0; 0 | 0; 0 |  |
| TCEB3C | Maternal | 0; 0 | 0; 0 |  |
| TFPI2 | Maternal | 0; 0 | 0; 0 |  |
| TP73 | Maternal | 0; 0 | 0; 0 |  |
| TRPM5 | Paternal | 0; 1 | 0; 0 |  |
| UBE3A | Maternal | 0; 0 | 0; 0 |  |
| VTRNA2-1 | Paternal | 0; 0 | 0; 0 |  |
| WT1 | Paternal | 11; 12 | 5; 23 |  |
| WT1-AS | Paternal | 0; 0 | 0; 0 |  |
| ZC3H12C | Paternal | 0; 0 | 0; 0 |  |
| ZDBF2 | Paternal | 0; 0 | 0; 0 |  |
| ZFAT | Paternal | 0; 0 | 0; 0 |  |
| ZFAT-AS1 | Paternal | 0; 0 | 0; 0 |  |
| ZFP90 | Paternal | 0; 0 | 0; 0 |  |
| ZIM2 | Paternal | 0; 0 | 0; 0 |  |
| ZNF215 | Maternal | 1; 0 | 0; 0 |  |
| ZNF396 | Paternal | 0; 0 | 0; 0 |  |
| ZNF597 | Maternal | 0; 0 | 0; 0 |  |
| **Mouse** | | | | |
| **Gene name** | **Expressed allele** | **WOS returns** brown fat; brown adipose | **PM returns** brown fat; brown adipose | **Returns meeting criteria** |
| AF313042 | Maternal | 0; 0 | 0; 0 |  |
| AF357341 | Maternal | 0; 0 | 0; 0 |  |
| AF357355 | Maternal | 0; 0 | 0; 0 |  |
| AF357359 | Maternal | 0; 0 | 0; 0 |  |
| AF357425 | Maternal | 0; 0 | 0; 0 |  |
| AF357426 | Maternal | 0; 0 | 0; 0 |  |
| AF357428 | Maternal | 0; 0 | 0; 0 |  |
| Air | Paternal | 169; 158 | 21; 74 |  |
| AK155734 | Maternal | 0; 0 | 0; 0 |  |
| Ampd3 | Maternal | 0; 0 | 0; 0 |  |
| Ano1 | Maternal | 0; 0 | 0; 0 |  |
| Aqp1 | Maternal | 0; 1 | 0; 1 |  |
| Asb4 | Maternal | 0; 0 | 0; 0 |  |
| Ascl2 | Maternal | 0; 0 | 0; 0 |  |
| Atp10a | Maternal | 0; 0 | 0; 0 |  |
| B830012L14Rik | Maternal | 0; 0 | 0; 0 |  |
| Calcr | Maternal | 0; 0 | 0; 0 |  |
| Cd81 | Maternal | 4; 4 | 1; 2 | x |
| Cdh15 | Paternal | 0; 0 | 0; 0 |  |
| Cdkn1c | Maternal | 2; 2 | 1; 2 | x |
| Chrac1 | Maternal | 0; 0 | 0; 0 |  |
| Commd1 | Maternal | 0; 0 | 0; 0 |  |
| Copg2 | Maternal | 0; 0 | 0; 0 |  |
| Dact2 | Maternal | 0; 0 | 0; 0 |  |
| Dcn | Maternal | 0; 3 | 0; 0 | x |
| Ddc | Paternal | 2; 2 | 0; 1 |  |
| Dhcr7 | Maternal | 1; 0 | 0; 0 |  |
| Dio3 | Paternal | 7; 13 | 5; 9 | x |
| Dio3os | Maternal | 1; 2 | 1; 2 | x |
| Dlk1 | Paternal | 5; 8 | 4; 13 | x |
| Fkbp6 | Paternal | 0; 0 | 0; 0 |  |
| Fthl17a | Paternal | 0; 0 | 0; 0 |  |
| Fthl17b | Paternal | 0; 0 | 0; 0 |  |
| Fthl17c | Paternal | 0; 0 | 0; 0 |  |
| Fthl17d | Paternal | 0; 0 | 0; 0 |  |
| Fthl17e | Paternal | 0; 0 | 0; 0 |  |
| Fthl17f | Paternal | 0; 0 | 0; 0 |  |
| Ftx | Paternal | 0; 0 | 0; 0 |  |
| Gab1 | Paternal | 1; 1 | 0; 1 |  |
| Galnt6 | Paternal | 0; 0 | 0; 0 |  |
| Gatm | Maternal | 1; 1 | 0; 1 | x |
| Gm35612 | Maternal | 0; 0 | 0; 0 |  |
| Gpr1 | Paternal | 2; 2 | 0; 1 |  |
| Gtl2 | Maternal | 1; 1 | 0; 0 |  |
| H13 | Maternal | 0; 0 | 0; 0 |  |
| H19 | Maternal | 4; 4 | 2; 1 | x |
| Htr2a | Maternal | 1; 1 | 0; 1 |  |
| Hymai | Paternal | 0; 0 | 0; 0 |  |
| Igf2 | Paternal | 4; 5 | 1; 4 |  |
| Igf2as | Paternal | 0; 0 | 0; 0 |  |
| Igf2r | Maternal | 1; 2 | 0; 2 |  |
| Impact | Paternal | 1,155; 1,137 | 109; 486 |  |
| Inpp5f V2 | Paternal | 0; 0 | 0; 0 |  |
| Ins1 | Paternal | 3; 5 | 1; 6 |  |
| Ins2 | Paternal | 1; 3 | 0; 3 |  |
| Jade1 | Paternal | 0; 0 | 0; 0 |  |
| Jpx | Paternal | 0; 0 | 0; 0 |  |
| Kcnk9 | Maternal | 0; 0 | 0; 1 |  |
| Kcnq1 | Maternal | 0; 0 | 0; 0 |  |
| Kcnq1ot1 | Paternal | 1; 1 | 0; 0 |  |
| Klf14 | Maternal | 0; 1 | 0; 0 |  |
| Magel2 | Paternal | 1; 1 | 0; 0 |  |
| Magi2 | Paternal | 0; 0 | 0; 0 |  |
| Mcts2 | Paternal | 0; 0 | 0; 0 |  |
| Mest | Paternal | 33; 39 | 0; 3 |  |
| Mir127 | Maternal | 0; 0 | 0; 0 |  |
| Mir134 | Maternal | 0; 0 | 0; 0 |  |
| Mir136 | Maternal | 0; 0 | 0; 0 |  |
| Mir154 | Maternal | 0; 0 | 0; 0 |  |
| Mir184 | Paternal | 0; 0 | 0; 0 |  |
| Mir296 | Paternal | 0; 0 | 0; 0 |  |
| Mir298 | Paternal | 0; 0 | 0; 0 |  |
| Mir337 | Maternal | 0; 0 | 0; 1 | x |
| Mir370 | Maternal | 0; 0 | 0; 1 |  |
| Mir376b | Maternal | 0; 0 | 0; 0 |  |
| Mir380 | Maternal | 0; 0 | 0; 0 |  |
| Mir410 | Maternal | 0; 0 | 0; 0 |  |
| Mir411 | Maternal | 0; 0 | 0; 0 |  |
| Mir431 | Maternal | 0; 0 | 0; 0 |  |
| Mirg | Maternal | 1; 1 | 0; 0 |  |
| Mkrn1-ps1 | Paternal | 0; 0 | 0; 0 |  |
| Mkrn3 | Paternal | 0; 1 | 0; 0 |  |
| Nap1l4 | Maternal | 0; 0 | 0; 0 |  |
| Nap1l5 | Paternal | 0; 0 | 0; 0 |  |
| Nctc1 | Paternal | 0; 0 | 0; 0 |  |
| Ndn | Paternal | 1; 0 | 0; 0 | x |
| Nespas | Paternal | 0; 0 | 0; 0 |  |
| Nlrp2 | Maternal | 0; 0 | 0; 1 |  |
| Nnat | Paternal | 1; 2 | 0; 3 | x |
| Pde10a | Maternal | 0; 2 | 0; 1 | x |
| Peg10 | Paternal | 1; 0 | 0; 0 |  |
| Peg12 | Paternal | 0; 0 | 0; 0 |  |
| Peg13 | Paternal | 0; 0 | 0; 0 |  |
| Peg3 | Paternal | 0; 0 | 0; 0 |  |
| Peg3os | Maternal | 0; 0 | 0; 0 |  |
| Phlda2 | Maternal | 0; 0 | 0; 0 |  |
| Plagl1 | Paternal | 1; 1 | 0; 0 |  |
| Platr20 | Paternal | 0; 0 | 0; 0 |  |
| Pon2 | Maternal | 3; 0 | 0; 0 |  |
| Pon3 | Maternal | 0; 0 | 0; 0 |  |
| Ppp1r9a | Maternal | 0; 0 | 0; 0 |  |
| Prkn | Maternal | 2; 1 | 2; 3 |  |
| Pwcr1 | Paternal | 0; 0 | 0; 0 |  |
| Qpct | Maternal | 0; 0 | 0; 0 |  |
| Rasgrf1 | Paternal | 0; 0 | 0; 0 |  |
| Rian | Maternal | 3; 0 | 0; 0 |  |
| Rtl1 | Paternal | 0; 0 | 0; 0 |  |
| Sfmbt2 | Paternal | 0; 0 | 0; 1 |  |
| Sgce | Paternal | 1; 0 | 0; 0 |  |
| Slc22a18 | Maternal | 0; 0 | 0; 0 |  |
| Slc22a2 | Maternal | 0; 0 | 0; 1 |  |
| Slc22a3 | Maternal | 2; 2 | 0; 0 |  |
| Slc38a4 | Paternal | 0; 0 | 0; 0 |  |
| Smoc1 | Paternal | 0; 0 | 0; 0 |  |
| Smoc2 | Maternal | 0; 0 | 0; 0 |  |
| Snrpn | Paternal | 1; 0 | 0; 0 |  |
| Snurf | Paternal | 0; 0 | 0; 0 |  |
| Snx14 | Paternal | 0; 0 | 0; 0 |  |
| Tfpi2 | Maternal | 0; 0 | 0; 0 |  |
| Tgfb1 | Maternal | 2; 2 | 1; 5 |  |
| Th | Maternal | 190; 239 | 11; 145 |  |
| Thbs2 | Maternal | 1; 2 | 0; 0 |  |
| Tnfrsf22 | Maternal | 0; 0 | 0; 0 |  |
| Tnfrsf23 | Maternal | 0; 0 | 0; 0 |  |
| Tnfrsf26 | Maternal | 0; 0 | 0; 0 |  |
| Trappc9 | Maternal | 0; 0 | 0; 0 |  |
| Tsix | Maternal | 0; 0 | 0; 0 |  |
| Tssc4 | Maternal | 0; 0 | 0; 0 |  |
| U2af1-rs1 | Paternal | 0; 0 | 0; 0 |  |
| Ube3a | Maternal | 0; 0 | 0; 0 |  |
| Usp29 | Paternal | 0; 0 | 0; 0 |  |
| Wt1 | Maternal | 11; 12 | 5; 23 |  |
| Xist | Paternal | 1; 2 | 0; 1 |  |
| Xlr3b | Maternal | 1; 1 | 1; 0 |  |
| Zcchc13 | Maternal | 0; 0 | 0; 0 |  |
| Zdbf2 | Paternal | 0; 0 | 0; 0 |  |
| Zfp127as | Maternal | 0; 0 | 0; 0 |  |
| Zfp264 | Paternal | 0; 0 | 0; 0 |  |
| Zfp64 | Paternal | 0; 0 | 0; 0 |  |
| Zim1 | Maternal | 0; 0 | 0; 0 |  |
| Zim2 | Maternal | 0; 0 | 0; 0 |  |
| Zim3 | Maternal | 0; 0 | 0; 0 |  |
| Zrsr1 | Paternal | 0; 0 | 0; 0 |  |
