# Supplementary material for: Mother’s warmth from maternal genes: genomic imprinting of brown adipose tissue
Source: Evol Med Public Health. 2023 Sep 29;11(1):379–85. doi: 10.1093/emph/eoad031 (PMC10621903; doi:10.1093/emph/eoad031)
Supplement: eoad031_suppl_Supplementary_Table_S2 [file eoad031_suppl_supplementary_table_s2.docx]

Supplementary Table 2. Results returned from searches using the Imprinted Gene and Parent of

Origin Effect Database

| **Gene (Transcriptional units as shown)** | **Human** | **Mouse** | **Expressed allele** | **WOS returns** brown fat; brown adipose | **PM returns**  brown fat; brown adipose | **Meeting criteria**  **(see citations in Table 1)** |
| --- | --- | --- | --- | --- | --- | --- |
| (4930524O08Rik, A19) both | O | I | P | 0; 0 | 0; 0 |  |
| (AS4) | O | I | P | 0; 0 | 0; 0 |  |
| (BB077283) | O | I | P | 0; 0 | 0; 0 |  |
| (BM117114) | O | I | P | 0; 0 | 0; 0 |  |
| (F7), Exon-1A both | I | I | P | 4; 2 | 0; 3 |  |
| (F7), GS-alpha both | I | I | M | 9; 16 | 6; 12 |  |
| (Mico1) | O | I | M | 0; 0 | 0; 0 |  |
| (Mico1os) | O | I | M | 0; 0 | 0; 0 |  |
| (Msuit1, AF313042) both | O | I | M | 0; 0 | 0; 0 |  |
| (Pec2) | O | I | P | 0; 0 | 0; 0 |  |
| (Pec3) | O | I | P | 0; 0 | 0; 0 |  |
| (Peg12) | O | I | P | 0; 0 | 0; 0 |  |
| (Peg13) | O | I | P | 0; 0 | 0; 0 |  |
| (PIHit) | O | I | P | 0; 0 | 0; 0 |  |
| (Rhox5) | O | I | P/M | 0; 0 | 0; 0 |  |
| (Tnfrsf22) | O | I |  | 0; 0 | 0; 0 |  |
| (Tnfrsf26) | O | I |  | 0; 0 | 0; 0 |  |
| (Xlr3b) | O | I | M | 1; 1 | 1; 0 |  |
| (Xlr4b) | O | I | M | 1; 1 | 1; 0 |  |
| (Xlr4c) | O | I | M | 0; 0 | 0; 0 |  |
| (Zim1) | O | I | M | 0; 0 | 0; 0 |  |
| (Zrsr1) | O | I | P | 0; 0 | 0; 0 |  |
| AIM1 (Aim1) | I | O | P | 0; 0 | 0; 0 |  |
| AIRN (Airn) | O | I | P | 0; 0 | 0; 0 |  |
| Anti-PEG11 (anti-Rtl1), anti-Rtl1 | O | I | M | 0; 0 | 0; 0 |  |
| Anti-PEG11 (anti-Rtl1), miR-127 | O | I | M | 0; 0 | 0; 0 |  |
| Anti-PEG11 (anti-Rtl1), miR-136 | O | I | M | 0; 0 | 0; 0 |  |
| Anti-PEG11 (anti-Rtl1), miR-431 | O | I | M | 0; 0 | 0; 0 |  |
| ASB4 (Asb4) | O | I | M | 0; 0 | 0; 0 |  |
| ASCL2 (Ascl2) | O | I | M | 0; 0 | 0; 0 |  |
| AXL (Axl) | O | I | M | 0; 0 | 0; 0 |  |
| BEGAIN (Begain) | O | I | P | 0; 0 | 0; 0 |  |
| BLCAP (Blcap) | I | I | M/P | 0; 0 | 0; 0 |  |
| C14MC (Mirg), C14MC (Mirg) | O | I | M | 0; 0 | 0; 0 |  |
| C14MC (Mirg), miR-134 | I | I | M | 0; 0 | 0; 0 |  |
| C14MC (Mirg), miR-154 | O | I | M | 0; 0 | 0; 0 |  |
| C14MC (Mirg), miR-376 | O | I | M | 0; 0 | 0; 0 |  |
| C14MC (Mirg), miR-376b | O | I | M | 0; 0 | 0; 0 |  |
| C14MC (Mirg), miR-380 | O | I | M | 0; 0 | 0; 0 |  |
| C14MC (Mirg), miR-410 | O | I | M | 0; 0 | 0; 0 |  |
| C14MC (Mirg), miR-411 | O | I | M | 0; 0 | 0; 0 |  |
| C19MC | I | O | P | 0; 0 | 0; 0 |  |
| CALCR (Calcr) | I | I | M | 0; 0 | 0; 0 |  |
| CD81 (Cd81) | O | I | M | 4; 4 | 1; 2 | x |
| CDH15 (Cdh15), 5' transcript (mouse) | O | I |  | 0; 0 | 0; 0 |  |
| CDKN1C (Cdkn1c) | I | I | M | 2; 2 | 1; 2 | x |
| COMMD1 (Commd1) | O | I | M | 0; 0 | 0; 0 |  |
| COPG2 (Copg2) | O | I | P(M)d | 0; 0 | 0; 0 |  |
| CPA4 (Cpa4) | I | O | M | 0; 0 | 0; 0 |  |
| DDC (Ddc), Exon1a transcript | O | I | P | 2; 2 | 0; 1 |  |
| DIO3 (Dio3) | O | I | P | 7; 13 | 5; 9 |  |
| DLGAP2 | I | O | P | 0; 0 | 0; 0 |  |
| DLK1 (Dlk1) | I | I | P | 5; 8 | 4; 13 |  |
| DLK1 downstream transcripts whole term | O | I | P | 0; 0 | 0; 0 |  |
| DNMT1 | I | O |  | 6; 7 | 2; 4 |  |
| FAM50B (Fam50b) | I | O | P | 0; 0 | 0; 0 |  |
| FAM50B-AS | I | O | P | 0; 0 | 0; 0 |  |
| GAB1 (Gab1) | O | I | P | 1; 1 | 0; 1 |  |
| GNAS |  |  |  | 5; 15 | 2; 9 | x |
| GNAS (Gnas), NESP55 | I | I | M | 1; 1 | 0; 0 | x |
| GNAS (Gnas), GNASXL | I | I | P | 0; 2 | 0; 1 | x |
| GPR1-AS (Zdbf2linc) both | I | I | P | 0; 0 | 0; 0 |  |
| GRB10 (Grb10) | I | I | M(P)c | 2; 2 | 0; 1 | x |
| H19 (H19) | I | I | M | 4; 4 | 2; 1 | x |
| H19 (H19), miR-675-3p | I | I |  | 0; 0 | 0; 0 |  |
| H19 (H19), miR-675-5p | I | I | M | 0; 0 | 0; 0 |  |
| HM13 (H13) | O | I | M | 0; 0 | 0; 0 |  |
| HTR2A (Htr2a) | O | I | M | 1; 1 | 0; 1 |  |
| HYMAI (Hymai) | I | O | P | 0; 0 | 0; 0 |  |
| IGF2 (Igf2), IGF2 (Igf2) | I | I | P | 4; 5 | 1; 4 |  |
| IGF2AS (Igf2as) | I | I | P | 0; 0 | 0; 0 |  |
| IGF2R (Igf2r) | O | I | M | 1; 2 | 0; 2 |  |
| IMPACT (Impact) | O | I | P | 1,155; 1,137 | 109; 486 |  |
| INPP5F_V2 (Inpp5f_v2) | I | I | P | 0; 0 | 0; 0 |  |
| INS (Ins2) | I | I | P | 16; 20 | 0; 4 |  |
| JADE1 (Jade1) | O | I | P | 0; 0 | 0; 0 |  |
| KCNK9 (Kcnk9) | I | I | M | 0; 0 | 0; 1 |  |
| KCNQ1 (Kcnq1) | I | I | M | 0; 0 | 0; 0 |  |
| KCNQ1DN | I | O | M | 0; 0 | 0; 0 |  |
| KCNQ1OT1 (Kcnq1ot1) | I | I | P | 1; 1 | 0; 0 |  |
| KLF14 (Klf14) | I | I | M | 0; 1 | 0; 0 |  |
| L3MBTL (L3mbtl) | I | O | P | 0; 0 | 0; 0 |  |
| MAGEL2 (Magel2) | I | I | P | 1; 1 | 0; 0 |  |
| MEG3 (Meg3) | I | I | M | 3; 2 | 0; 1 |  |
| MEG8 (Rian), MEG8 (Rian) | I | I | M | 0; 0 | 0; 0 |  |
| MEG8 (Rian), (MBII-19) | O | I | M | 0; 0 | 0; 0 |  |
| MEG8 (Rian), (MBII-426) | O | I | M | 0; 0 | 0; 0 |  |
| MEG8 (Rian), (MBII-49) | O | I | M | 0; 0 | 0; 0 |  |
| MEG8 (Rian), (MBII-78) | O | I | M | 0; 0 | 0; 0 |  |
| MEG8 (Rian), 14q(0) | O | I | M | 0; 0 | 0; 0 |  |
| MEG8 (Rian), 14q(I) (MBII-48) | O | I | M | 0; 0 | 0; 0 |  |
| MEG8 (Rian), 14q(II) (MBII-343) | O | I | M | 0; 0 | 0; 0 |  |
| MEG8 (Rian), miR-370 | O | I | M | 0; 1 | 0; 1 |  |
| MEST (Mest) | I | I | P | 33; 39 | 0; 3 |  |
| MESTIT1 | I | O | P | 0; 0 | 0; 0 |  |
| MIMT1 | I | O | P | 0; 0 | 0; 0 |  |
| MIR337 (Mir337) | I | I | M | 0; 0 | 0; 1 | x |
| MIRN184 (Mirn184) | O | I | P | 0; 0 | 0; 0 |  |
| MIRN335 (Mirn335) | O | I |  | 0; 0 | 0; 0 |  |
| MKRN3 (Mkrn3) | I | I | P | 0; 1 | 0; 0 |  |
| NAA60, NAA60 isoform 1 | I | O | M | 0; 0 | 0; 0 |  |
| NAP1L5 (Nap1l5) | I | I | P | 0; 0 | 0; 0 |  |
| NDN (Ndn)(necdin) | I | I | P | 1; 0 | 0; 0 | x |
| NNAT (Nnat)(neuronatin) | I | I | P | 1; 2 | 0; 3 | x |
| NPAP1 | I | O | NK | 0; 0 | 0; 0 |  |
| PDE10A (Pde10a) | O | I | M | 0; 2 | 0; 1 | x |
| PEG10 (Peg10) | I | I | P | 1; 0 | 0; 0 |  |
| PEG3 (Peg3) | I | I | P | 0; 0 | 0; 0 |  |
| PHACTR2 (Phactr2) | I | I | M | 0; 0 | 0; 0 |  |
| PHLDA2 (Phlda2) | I | I | M | 0; 0 | 0; 0 |  |
| PLAGL1 (Plagl1) | I | I | P | 1; 1 | 0; 0 |  |
| PPP1R9A (Ppp1r9a) | O | I | M | 0; 0 | 0; 0 |  |
| PSIMCT-1^a^ (Mcts2^b^) | I | I | P | 0^a^, 0^b^; 0^a^, 0^b^ | 0^a^, 0^b^; 0^a^, 0^b^ |  |
| RASGRF1 (Rasgrf1) | O | I | P | 0; 0 | 0; 0 |  |
| RB1 (Rb1), RB1 | I | O | M | 9; 14 | 1; 6 | x |
| RB1 (Rb1), RB1 2B | I | O | P | 0; 0 | 0; 0 |  |
| RNU5D-1 | I | O | P | 0; 0 | 0; 0 |  |
| RTL1^c^ (Rtl1; PEG11^d^) | I | I | P | 0^c^, 0^d^; 0^c^, 0^d^ | 0^c^, 0^d^; 0^c^, 0^d^ |  |
| SANG | I | I | P | 98; 100 | 0; 5 |  |
| Nespas | I | I | P | 0; 0 | 0; 0 |  |
| SANG (Nespas), miR-296 | I | I | P | 0; 0 | 0; 0 |  |
| SANG (Nespas), miR-298 | I | I | P | 0; 0 | 0; 0 |  |
| SFMBT2 (Sfmbt2) | O | I | P | 0; 0 | 0; 1 |  |
| SFMBT2 (Sfmbt2), Intron 10 miRNA cluster. all | O | I |  | 0; 0 | 0; 1 |  |
| SLC22A18 (Slc22a18) | I | I | M | 0; 0 | 0; 0 |  |
| SLC22A2 (Slc22a2) | O | I | M | 0; 0 | 0; 1 |  |
| SLC22A3 (Slc22a3) | O | I | M | 2; 2 | 0; 0 | x |
| SLC38A4 (Slc38a4) | O | I | P | 0; 0 | 0; 0 |  |
| SNURF-SNRPN, IPW | I | I | P | 0; 0 | 0; 0 |  |
| SNURF-SNRPN, SNORD107 (MBII-436) | I | I | P | 0; 0 | 0; 0 |  |
| SNURF-SNRPN, SNORD115@ | I | I | P | 0; 0 | 0; 0 |  |
| SNURF-SNRPN, SNORD116@ (Pwcr1) | I | I | P | 0; 0 | 0; 0 |  |
| SNURF-SNRPN, SNORD64 (MBII-13) | I | I | P | 0; 0 | 0; 0 |  |
| SNURF-SNRPN, SNRPN (Snrpn) | I | I | P | 1; 0 | 0; 0 |  |
| SNURF-SNRPN, SNURF (Snurf) | I | I | P | 0; 0 | 0; 0 |  |
| SNURF-SNRPN, UBE3A-AS | I | I | P | 0; 0 | 0; 0 |  |
| SNURF-SNRPN, SNORD108 | I | O | P | 0; 0 | 0; 0 |  |
| SNURF-SNRPN, SNORD109A | I | O | P | 0; 0 | 0; 0 |  |
| SNURF-SNRPN, SNORD109B | I | O | P | 0; 0 | 0; 0 |  |
| TFPI2 (Tfpi2) | I | I | M | 0; 0 | 0; 0 |  |
| TH (Th) | O | I | M | 190; 239 | 11; 145 |  |
| TP73 (Trp73) | I | O | M | 0; 0 | 0; 0 |  |
| TSIX (Tsix) | O | I | M | 0; 0 | 0; 0 |  |
| TSSC4 (Tssc4) | O | I | M | 0; 0 | 0; 0 |  |
| UBE3A (Ube3a) | I | I | M | 0; 0 | 0; 0 |  |
| USP29 (Usp29)e | O | I | P | 0; 0 | 0; 0 |  |
| WT1-Alt transcript (Wt1), Check name on entry. | I | O | P | 11; 12 | 5; 23 |  |
| XIST (Xist) | O | I | P | 1; 2 | 0; 1 |  |
| ZDBF2 (Zdbf2) | I | I | P | 0; 0 | 0; 0 | x |
| ZIM2 (Zim2) | I | I | P(M)d | 0; 0 | 0; 0 |  |
| ZIM3 (Zim3) | O | I | M | 0; 0 | 0; 0 |  |
| ZNF264 (Zfp264) | O | I | P | 0; 0 | 0; 0 |  |
| ZNF597 (Zfp597) | I | O | M | 0; 0 | 0; 0 |  |
